# The Taspase1/Myosin1f-axis regulates filopodia dynamics

**DOI:** 10.1016/j.isci.2022.104355

**Published:** 2022-05-05

**Authors:** Astrid Hensel, Paul Stahl, Lisa Moews, Lena König, Rutuja Patwardhan, Alexander Höing, Nina Schulze, Perihan Nalbant, Roland H. Stauber, Shirley K. Knauer

**Affiliations:** 1 Department of Molecular Biology II, Center of Medical Biotechnology (ZMB), University Duisburg-Essen, 45141 Essen, Germany; 2 Department of Molecular Cell Biology, Center of Medical Biotechnology (ZMB), University Duisburg-Essen, 45141 Essen, Germany; 3 Imaging Center Campus Essen (ICCE), Center of Medical Biotechnology (ZMB), University Duisburg-Essen, 45141 Essen, Germany; 4 Department of Molecular and Cellular Oncology/ENT, University Mainz Medical Center, 55131 Mainz, Germany

**Keywords:** Biological sciences, Cell biology, Immunology

## Abstract

The unique threonine protease Tasp1 impacts not only ordered development and cell proliferation but also pathologies. However, its substrates and the underlying molecular mechanisms remain poorly understood. We demonstrate that the unconventional Myo1f is a Tasp1 substrate and unravel the physiological relevance of this proteolysis. We classify Myo1f as a nucleo-cytoplasmic shuttle protein, allowing its unhindered processing by nuclear Tasp1 and an association with chromatin. Moreover, we show that Myo1f induces filopodia resulting in increased cellular adhesion and migration. Importantly, filopodia formation was antagonized by Tasp1-mediated proteolysis, supported by an inverse correlation between Myo1f concentration and Tasp1 expression level. The Tasp1/Myo1f-axis might be relevant in human hematopoiesis as reduced Tasp1 expression coincided with increased Myo1f concentrations and filopodia in macrophages compared to monocytes and vice versa. In sum, we discovered Tasp1-mediated proteolysis of Myo1f as a mechanism to fine-tune filopodia formation, inter alia relevant for cells of the immune system.

## Highlights

• Myosin1f is a nucleo-cytoplasmic shuttle protein temporarily located in the nucleus
• Myosin1f induces filopodia resulting in increased cellular adhesion and migration
• The protease Taspase1 cleaves Myosin1f, thereby impairing its function
• Taspase1 and Myosin1f inversely correlate in immune cell differentiation

## Graphical abstract

**Figure:**
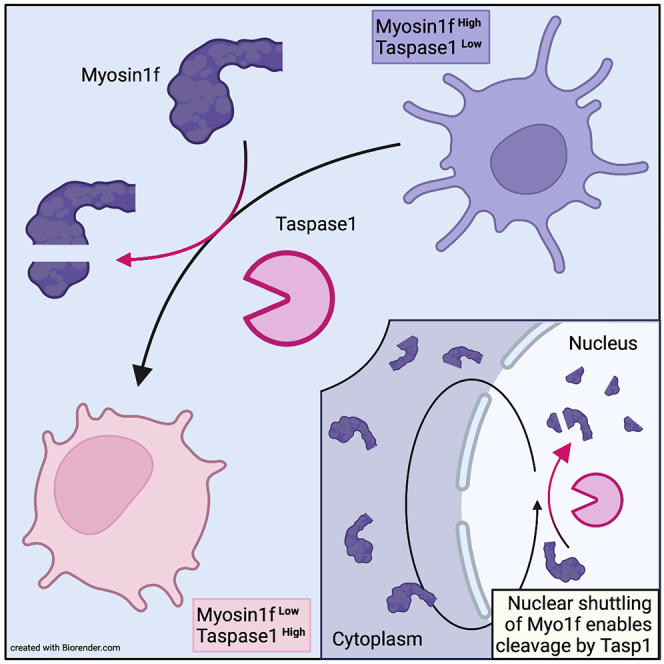

## Introduction

Taspase1 (Tasp1) or Threonine Aspartase 1 was discovered as the protease responsible for the proteolytic activation of mixed-lineage leukemia 1 (MLL1) protein (Hsieh et al., 2003a, 2003b). Further verified Tasp1 targets comprise MLL2, MLL4 (Hsieh et al., 2003b; Takeda et al., 2006), the transcription factors TFIIA (Zhou et al., 2006; Schrenk et al., 2018), ALF (Høiby et al., 2004), upstream stimulatory factor 2 (USF2) (Bier et al., 2011b) as well as REV3L, the catalytic subunit of DNA polymerase ζ (Wang et al., 2020). Subsequently, refining Tasp1’s consensus recognition sequence to Q[F,I,L,V]D↓GXDD (Bier et al., 2011b) was a prerequisite for the identification of additional cellular substrates. A genome-wide bioinformatic screen has identified 27 putative Tasp1 target proteins, comprising the hitherto experimentally verified and published as well as previously unknown substrates (Bier et al., 2011b).

Among the latter is the protein Myosin1f (Myo1f), a monomeric class I myosin, which belongs to the subgroup of unconventional myosins (Crozet et al., 1997) that do not form filaments unlike conventional myosin (Kalhammer and Bähler, 2000). Myosins are actin-based, ATP-dependent motor proteins characterized by a tripartite structure: the middle ‘neck’ domain interconnects the conserved N-terminal motor domain and the highly divergent C-terminal ‘tail’ domain.

Class-I myosins are known to execute diverse biological functions. They are involved in membrane dynamics and actin organization, thus affecting cell migration, endo-, exo- and phagocytosis (Mermall et al., 1998; Maravillas-Montero and Santos-Argumedo, 2012). Myo1f is expressed predominantly in the spleen, mesenteric lymph nodes, thymus and lung, and in particular in certain cells of the mammalian immune system including natural killer cells, macrophages (Mφ), dendritic cells and neutrophils (Kim et al., 2006). Myo1f-deficient neutrophils show a severely impaired migration rate on fibronectin and Myo1f-deficient mice are more susceptible to infection (Kim et al., 2006). Furthermore, Myo1f is required for neutrophil migration in a 3D environment during acute inflammation (Salvermoser et al., 2018). However, the exact molecular function of Myo1f in immune cell motility and thus the innate immune response still remains to be elucidated.

Myosin-X, another unconventional myosin, was revealed to stimulate the formation and elongation of filopodia (Berg et al., 2000; Berg and Cheney, 2002; Zhang et al., 2004; Bohil et al., 2006; Tokuo et al., 2007). Filopodia are thin, finger-like protrusions of the plasma membrane that contain tightly packed parallel bundles of actin filaments (Small, 1988). They are typically less than 10 μm long but can extend up to 70 μm, depending on the cell type (Mogilner and Rubinstein, 2005; Schäfer et al., 2011).

Here, we demonstrate that Myo1f induces the formation of filopodia-like plasma membrane protrusions which lead to increased cell adhesion and migration properties. Moreover, we discovered that Myo1f is a *bona fide* Tasp1 substrate and we also provide valuable hints for the physiological relevance of this proteolysis during human hematopoiesis.

## Results

### Myo1f is a *bona fide* Tasp1 substrate

**Figure 1:**
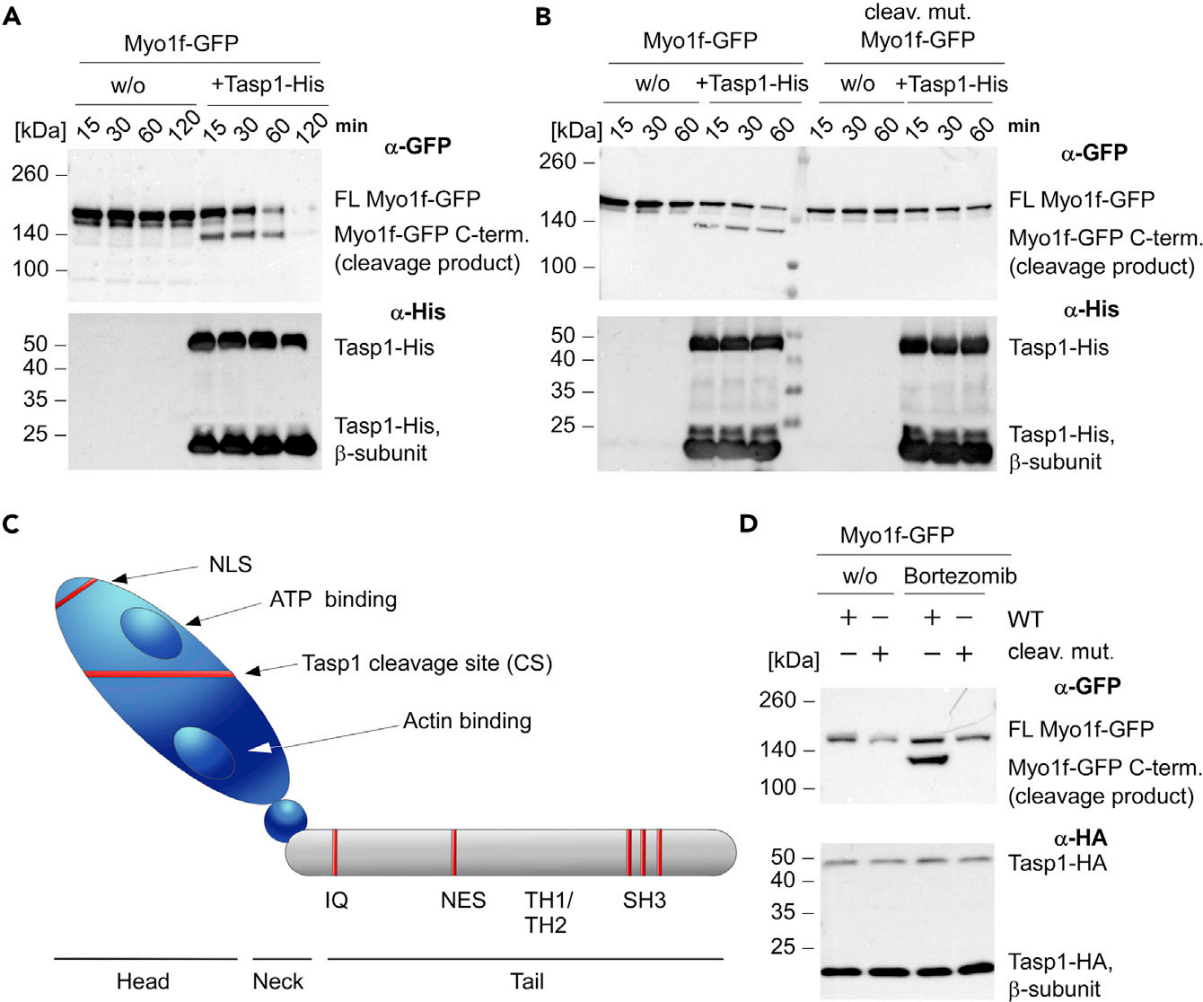
Myo1f is a *bona fide* substrate of Tasp1 (A/B) A *semi-in vitro* Tasp1 substrate cleavage assay was employed to verify the predicted Tasp1 cleavage site of Myo1f. 293T cells were transfected with plasmids encoding Myo1f-GFP or the cleavage site-deficient mutant Myo1f-^245^AA^246^-GFP. 24 h after transfection, cell lysates were incubated with or without 10 μM recombinant Tasp1-His at 37°C for the indicated time periods. The assay was performed without (A) or with (B) a two-fold concentrated protease inhibitor mix. Immunoblot analysis revealed the appearance of the C-terminal cleavage product (calculated molecular weight: 124 kDa) only in case of wild-type Myo1f and Tasp1-His presence confirming a Tasp1-His mediated cleavage of full-length (FL) Myo1f-GFP. (C) Schematic tripartite structure of Myo1f comprising a head (blue oval), a neck (blue sphere) and a tail (gray) domain. Indicated are the proposed nuclear localization signal (NLS) and the Tasp1 cleavage site (CS), the ATP and actin binding domains located within the head domain, and the nuclear export signal (NES)-bearing region (red) arranged between the IQ calmodulin-binding motif, the motor and tail homology 1 and 2 (TH1/TH2) and src homology 3 domains as part of the tail. (D) 293T cells co-expressing the indicated Myo1f variants and Tasp1-HA were treated with 50 nM bortezomib. Immunoblotting of whole cell extracts revealed a lower migrating band only for WT Myo1f-GFP after proteasome inhibition.

As endogenous Myo1f is rarely detectable in non-immune cells, we first employed the full-length open reading frame of *MYO1F*, encoding a protein of 1098 amino acids and 125 kDa, as a GFP fusion for mammalian expression. As an important control, we also generated a non-cleavable Myo1f mutant, in which the Tasp1 consensus cleavage site was deleted by a ^245^DG^246^ to alanine mutation (Myo1f-^245^AA^246^-GFP). A semi-*in vitro* Tasp1 substrate cleavage assay was established to investigate a putative Tasp1-mediated Myo1f cleavage. Briefly, 293T cells with neglectable endogenous Tasp1 expression were transfected with the respective Myo1f constructs. Then cell lysates were incubated with recombinant His-tagged Tasp1, resulting in a decreased amount of full-length Myo1f-GFP and the appearance of a lower migrating molecular species consistent with the calculated molecular weight (124 kDa) of the C-terminal Myo1f-GFP cleavage product (Figure 1A).

However, following prolonged incubation with recombinant Tasp1-His, both Myo1f-GFP species disappeared in a time-dependent manner, whereas the full-length Myo1f in the untreated samples remained stable over time. Thus, we repeated the assay in presence of a two-fold concentrated protease inhibitor mix, which indeed allowed the stabilization of at least the C-terminal Myo1f-fragment against proteolytic degradation over time (Figure 1B). Of note, Tasp1 itself is not inhibited by common protease inhibitors (Hsieh et al., 2003a). Moreover, this setup enabled us to confirm the predicted cleavage site in Myo1f, as we could not detect any cleavage product of the Myo1f-^245^AA^246^-GFP mutant, even after prolonged incubation with recombinant His-Tasp1 (Figure 1B). We further set out to verify proteolytic cleavage in a more physiological environment. Therefore, we analyzed 293T cell lysates with ectopic co-expression of Myo1f-GFP or the cleavage-site-deficient mutant and Tasp1-HA in the presence or absence of 50 nM bortezomib, a specific inhibitor of the 26S proteasome (Adams et al., 1999). Again, we could detect the C-terminal Tasp1 cleavage product only for the wild-type protein, but not the mutant (Figure 1D), further substantiating Myo1f as a *bona fide* Tasp1-substrate. Notably, in absence of the inhibitor, the C-terminal cleavage fragment was hardly detectable. As the cleavage is suggested to remove the ATP binding site pivotal for ATPase activity of Myo1f, the remaining Myo1f likely represents a non-functional motor protein destined for proteasomal degradation.

### Myo1f is a nucleo-cytoplasmic shuttle protein

**Figure 2:**
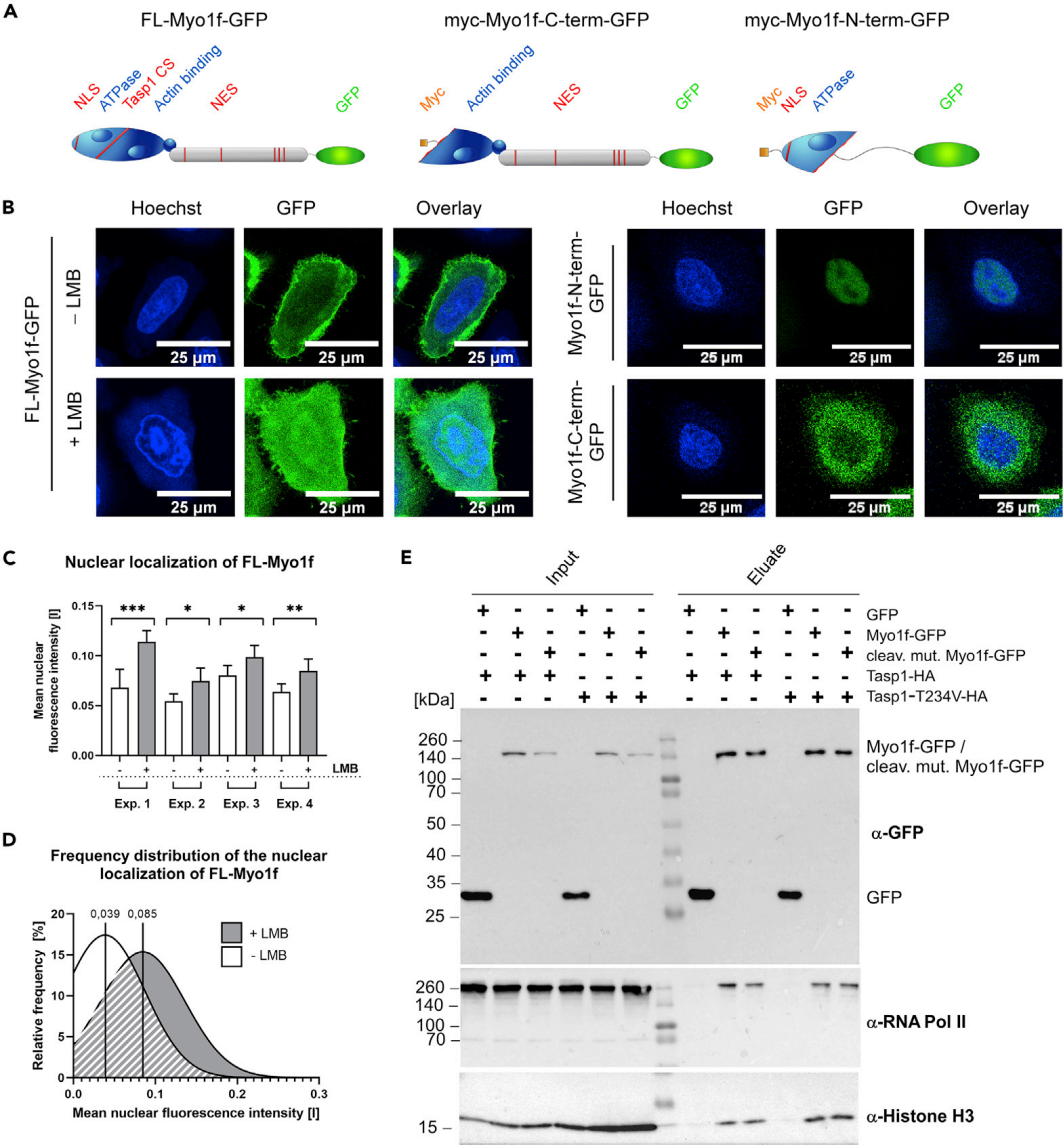
Myo1f is a nucleo-cytoplasmic shuttle protein (A) Schematic presentation of full-length Myo1f-GFP (left) and myc-/GFP-labeled Myo1f-truncations representing the N- (middle) and the C-terminal Tasp1 cleavage product (right). (B) Confocal fluorescence microscopy images showing the subcellular localization of Myo1f and truncated variants in HeLa cells. Full length Myo1f-GFP exhibited a predominantly cytosolic localization, but exposure to Leptomycin B (LMB) results in a shift to an increased nuclear localization (left panel). In contrast, the N-terminal fragment clearly localized to the nucleus, whereas the C-terminal fragment was exclusively cytoplasmic (right panel). Scale bars 25 μm. (C) Quantification of the intracellular localization of full-length Myo1f-GFP using the software “CellProfiler3” (Schindelin et al., 2012). Mean nuclear fluorescence intensity between 500 nm and 550 nm was measured in ROIs defined by Hoechst staining. Error bars represent the CI 95%, asterisks indicate statistical significance of mean difference assessed by t-test (∗: p ≤ 0.05; ∗∗: p ≤ 0.01; ∗∗∗: p ≤ 0.001). (D) Gaussian fit of the frequency distributions of measured mean nuclear fluorescence intensities further substantiated the increased nuclear localization upon LMB treatment, as two distinct populations become apparent. n = 2,572 cells from 4 independent experiments. (E) Myo1f-GFP and Myo1f-^245^AA^246^-GFP interact with RNA polymerase II and Histone H3. Co-immunoprecipitation of GFP-tagged Myo1f-variants from 293T chromatin fractions as detected by immunoblot analysis. Input = chromatin fraction; eluate, 40× concentrated. See also Figures S1 and S2.

However, it was still unclear how proteolytic processing and the accompanying removal of the ATP binding site on Myosin’s head domain may affect the cytosolic and membrane-associated localization of Myo1f. Therefore, localization of full-length Myo1f-GFP and GFP-fusions of myc-tagged Myo1f fragments representing the cleavage products resulting from Tasp1-mediated proteolysis was investigated with confocal fluorescence microscopy (Figure 2A). Here, the N-terminal Tasp1 cleavage fragment (myc-Myo1f-N-term-GFP, 55 kDa) exclusively showed nuclear accumulation, whereas the respective C-terminal cleavage construct (myc-Myo1f-C-term-GFP, 124 kDa), was solely localized in the cytoplasm (Figure 2B, right panel). These results prompted us to screen the Myo1f protein sequence for the presence of intracellular transport signals, in particular nuclear localization (NLS) and nuclear export signals (NES). First, the web-based motif predictor tool “cNLS mapper” (Kosugi et al., 2009) predicted a bipartite NLS in the extreme N-terminus of Myo1f and at least 5 potential nuclear export mediating sequences in the C-terminal portion of the protein were identified by *in silico* analysis with “NES Finder 0.2” and “LocNES” (Xu et al., 2015) (Figure S1). Thus, we decided to make use of the general nuclear export inhibitor Leptomycin B (LMB) irreversibly inhibiting the export receptor Exportin1/Crm1. Indeed, for full length Myo1f-GFP, which predominantly localizes to the plasma membrane and the cytoplasm in untreated cells, we could detect a partial, but robust nuclear accumulation in response to treatment with 5 nM LMB for 3 h (Figure 2B, left panel). This could be quantified in four independent experiments, in terms of a significant increase in the mean nuclear fluorescence intensity (Figure 2C) as well as a clear shift in the respective frequency distribution (Figure 2D). Moreover, the subcellular distribution pattern of Myo1f-GFP and its truncations could be confirmed by subcellular protein fractionation. Likewise, LMB treatment results in an enrichment of Myo1f-GFP in the nuclear protein extracts (Figure S2).

The potential of Myo1f to access the nucleus might be directly linked to its cleavage by the predominantly nuclear/nucleolar Tasp1, enabling optimal access of the protease to its substrates within the same cellular compartment. Moreover, its characterization as a nucleo-cytoplasmic shuttle protein places Myo1f in good company with further equally classified unconventional myosins, such as Myosin1C (Nevzorov et al., 2018).

To further confirm our results and to elucidate a possible functional role for the nuclear Myo1f, we performed co-immunoprecipitations from chromatin fractions. Precipitated full length Myo1f-GFP was found to be associated with RNA polymerase II and the histone protein H3 (Figure 2E). It should also be pointed out that the cleavage fragment of Myo1f is not detectable here as in this approach no bortezomib was added to prevent it from proteasomal degradation.

Previous studies described regulatory functions for nuclear myosins in transcriptional control and in positioning of chromosomes in chromosome territories or transcription factories (Philimonenko et al., 2004; Kulashreshtha et al., 2016). Based on our findings, a similar task in the nucleus may be considered for Myo1f. Of note, not only wild-type Myo1f-GFP, but also the variant with a mutated Tasp1-cleavage site was bound to chromatin and associated with the same nuclear proteins to a comparable extent (Figure 2E). Moreover, association of Myo1f with active chromatin occurs regardless of whether WT Tasp1-HA is co-expressed or the proteolytically inactive Tasp1 mutant Tasp1-T234V-HA. This suggests that cleavage of Myo1f is not a prerequisite for its nuclear localization, which is in line with our microscopic data.

### Myo1f induces filopodia formation

**Figure 3:**
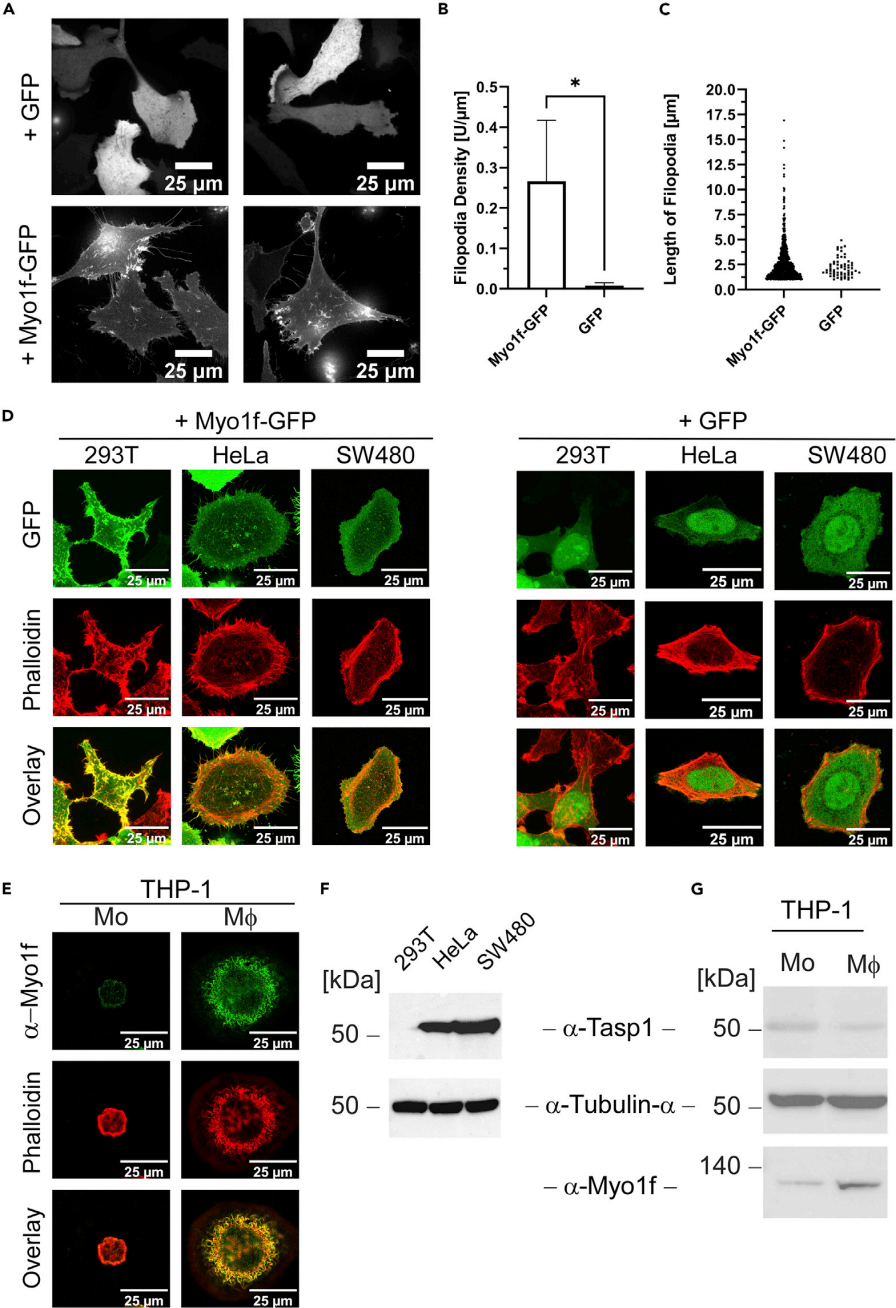
Myo1f-GFP induces filopodia-like membrane protrusions (A) Myo1f-GFP expressing HeLa cells are characterized by increased filopodia formation as detected by spinning disc confocal microscopy. Scale bars 25 μm. (B) FiloQuant analysis of relative filopodia density (filopodia number per μm cell surface) in HeLa cells expressing Myo1f-GFP compared to GFP expressing control cells. Statistical significance was assessed by t-test with a total of n = 100 cells/group in 3 independent experiments (∗: p < 0,05). Error bars represent the standard deviation. (C) FiloQuant measurement of filopodia longer than 1 μm in HeLa cells expressing Myo1f-GFP compared to GFP expressing control cells. n = 100 cells/group from 3 independent experiments. (D) Formation of Myo1f-GFP-induced cell protrusions containing filamentous F-actin in different adherent cell lines. 293T-, HeLa- and SW480 cells were transfected with either Myo1f-GFP (left panel) or GFP (right panel) and fixed 24 h later. F-actin was stained with rhodamine-conjugated phalloidin (red) and cells were analyzed by laser scanning confocal microscopy. Scale bars 25 μm. (E) THP-1 monocytes (Mo) were differentiated into macrophage-like cells (THP-1 macrophages, Mφ) by PMA treatment and analyzed by immunostaining. Endogenous Myo1f of THP-1 macrophages localizes to cell protrusions together with filamentous actin whereas THP-1 monocytes show comparatively less endogenous Myo1f and no filopodia. Scale bars 25 µm. (F) Immunoblot showing varying Tasp1 expression in different cell lines. (G) Decreased Tasp1 expression coincides with increased full-length Myo1f protein levels during differentiation from monocytes (Mo) into macrophages (Mφ) as demonstrated by immunoblot analysis. See also Figures S3 and S4.

To understand the biological effects of Myo1f cleavage, we aimed to gain more insights into its general cellular functions. Various cell lines (293T, HeLa, SW480) were transfected with Myo1f-GFP and analyzed by confocal microscopy. Myo1f-GFP was predominantly localized at the plasma membrane and in the cytoplasm of transfected cells (Figures 3A and 3D). Interestingly, expression of Myo1f-GFP (Figures 3A and 3D, left panel) led to impressive formation of thin plasma membrane protrusions reminiscent of filopodia. Staining with phalloidin revealed that F-actin and Myo1f-GFP clearly co-localized at the rim of the cell and within the membrane protrusions, indicating that the Myo1f-provoked structures are indeed filopodia (Figure 3D, left panel). Filopodia quantification with FiloQuant software (Jacquemet et al., 2017) showed a significant increase in relative filopodia density of Myo1f-GFP expressing HeLa cells compared to GFP expressing control cells (Figure 3B), demonstrating that the observed effect can be attributed to Myo1f overexpression. Moreover, only Myo1f-GFP expressing cells display filopodia longer than 5 μm (Figure 3C).

The extent of Myo1f-containing cell protrusions varies widely among the three different cell lines (Figure 3D): The non-cancerous 293T cell line exhibited the highest incidence of cell protrusions, whereas the cancerous cell lines HeLa and SW480 revealed a less and the least pronounced effect, respectively. To test whether differences in the Tasp1 expression level might account for this varying abundance and size of filopodia, we analyzed the different cell lines for endogenous protein amounts by immunoblot (Figure 3F). Indeed, Tasp1 was undetectable in 293T cells, whereas the 50 kDa proenzyme could be detected in lysates of HeLa cells and the highest expression level was found in SW480 cells.

As Myo1f is referred to be mostly expressed in innate leukocytes, we next compared THP-1 cells differentiated with PMA (phorbol 12-myristate 13-acetate) into macrophage-like cells (THP-1 macrophages) with their direct precursors - THP-1 monocytes. During the maturation of monocytes into macrophages, cells undergo distinct morphological changes. In particular, they enlarge, become adherent and develop membrane ruffles and filopodia. Treatment with PMA indeed induced such phenotypic alterations of THP-1 cells. Immunostaining of endogenous Myo1f revealed that it is localized in the filopodia of THP-1 macrophages. In THP-1 monocytes, lower amounts of endogenous Myo1f were detected and no Myo1f-containing cell protrusions were observed (Figure 3E). Likewise, immunoblot analyses demonstrated a reduced expression of full length Myo1f in THP-1 monocytes compared to macrophages in which Tasp1 concentration was decreased (Figure 3G).

The small Rho GTPase Cdc42, has been shown to control the formation of actin based filopodia and dynamic cell adhesions called focal complexes to the extracellular matrix (Nobes and Hall, 1995). Thus, we next investigated a potential interplay of Myo1f and Cdc42 in filopodia formation, by using wild-type mCherry-Cdc42 and a constitutively active mutant thereof, mCherry-Cdc42-Q61L. In the latter one, a Q61 to L amino acid substitution results in the loss of the intrinsic GTPase activity thereby preventing inactivation (Krengel et al., 1990; Nalbant et al., 2004). Both Cdc42 variants are at least partially co-localized with Myo1f-GFP at the plasma membrane and at the base of filopodia (Figure S3A). These findings were further substantiated by co-immunoprecipitation of Myo1f and the constitutively active form of Cdc42 (Figure S3B).

### Cleavage of Myo1f by Tasp1 antagonizes filopodia formation

**Figure 4:**
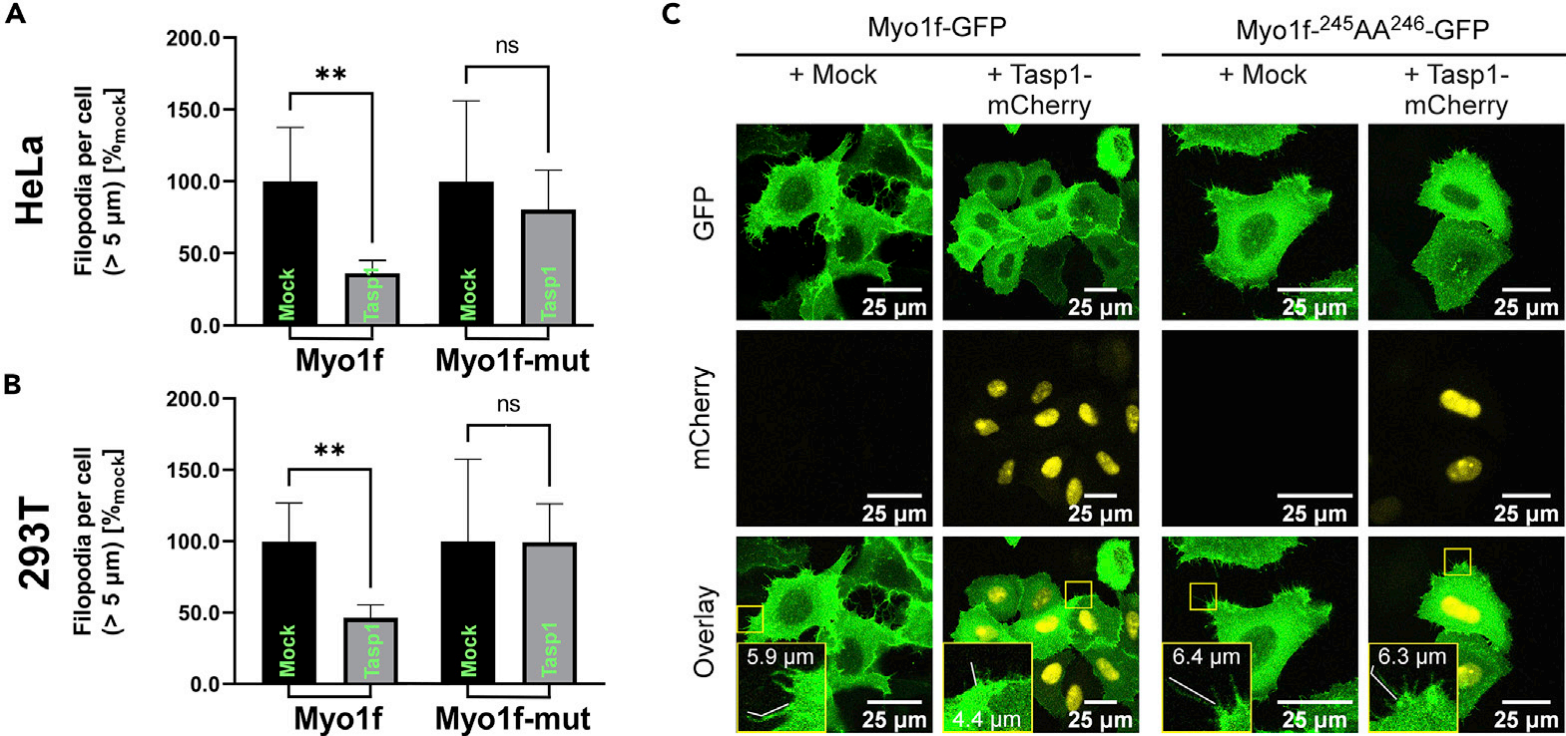
Tasp1 co-expression reduces filopodia in cells expressing Myo1f but not in cells expressing a non-cleavable Myo1f mutant (A/B) Counting of filopodia longer than 5 μm in HeLa (A) and 293T (B) cells expressing Myo1f- or Myo1f-^245^AA^246^-GFP together with either Tasp1-mCherrry (n = 83/n = 87) or a mock plasmid (n = 114/n = 94); filopodia counts per cell were normalized to the respective control cells without Tasp1 co-expression. Significance was analyzed by an unpaired t-test (∗∗: p ≤ 0.01; ns: not significant). Error bars indicate the standard deviation. (C) Representative confocal microscopy images of cells expressing the indicated constructs. Scale bars 25 μm.

Next, we analyzed whether Tasp1’s proteolytic cleavage activity is responsible for the observed inverse correlation of Tasp1 expression and the amount of Myo1f-provoked filopodia. Therefore, we tested whether Tasp1-mCherry co-expression affects Myo1f-GFP-induced filopodia formation. An uncleavable Myo1f mutant (Myo1f-^245^AA^246^-GFP) served as control. Filopodia amount and length were quantified in fluorescent confocal images of a sufficient number of HeLa and 293T cells (n > 80) expressing the respective Myo1f variant with or without Tasp1-mCherry. Of note, two classes of filopodia were categorized according to length: < 5 μm and > 5 μm. The amount of filopodia smaller than 5 μm seemed to be stable within each Myo1f expressing cell line and independent of Tasp1 co-expression. Therefore, filopodia longer than 5 μm were quantified and assessed by pairwise comparison via t-test among the cells expressing the same Myo1f variant with or without Tasp1 co-expression. Indeed, in Myo1f-GFP-expressing cells the number of filopodia longer than 5 μm was significantly reduced by Tasp1-mCherrry co-expression in both Hela- and 293T cells (Figure 4). Interestingly, we observed that expression of the uncleavable Myo1f mutant (Myo1f-^245^AA^246^-GFP) also promotes filopodia formation (Figure S4), underlining the relevance of unprocessed, full-length Myo1f for the promotion and/or maintenance of distinct cell protrusions. But in this case, the presence of Tasp1-mCherry did not result in a perturbed filopodia formation (Figure 4). This indicates that the inverse correlation of the cellular Tasp1 level and Myo1f-induced filopodia is indeed causally linked to the specific proteolysis of Myo1f. The latter is supposed to result in the elimination of the ATP binding site located in the head domain (Figure 1C). As such, it is conceivable that after the potential to produce mechanical energy is lost by Tasp1 cleavage, the ability of Myo1f to initiate or elongate cell protrusions decreases. This might in turn account for the observed inverse correlation of endogenous Tasp1 levels and the capability of Myo1f to efficiently induce, stabilize or elongate filopodia formation.

**Figure 5:**
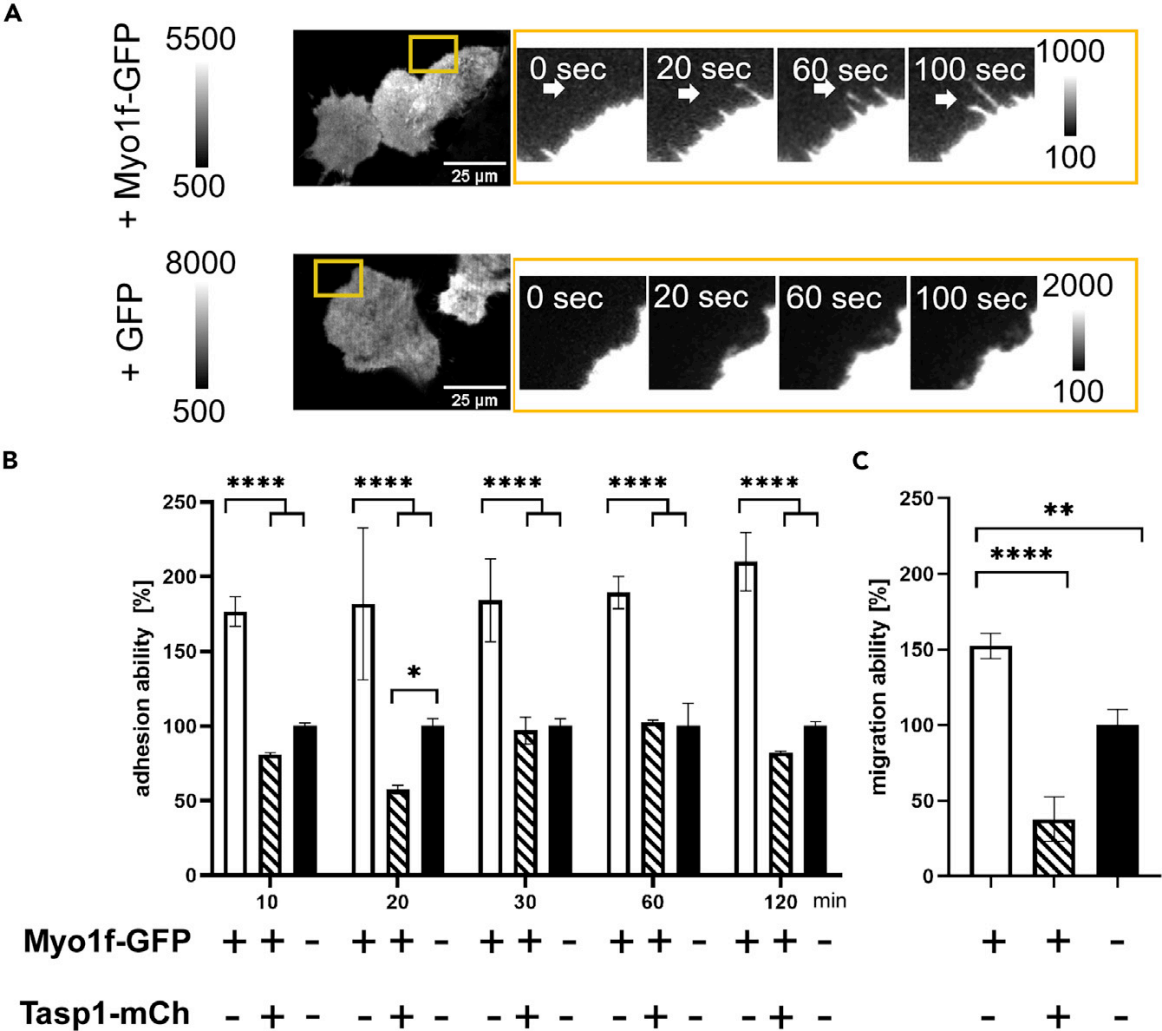
Myo1f facilitates cellular adhesion and migration (A) Myo1f-induced filopodia actively sense their environment. Representative TIRF images of cells overexpressing Myo1f-GFP or GFP control. Yellow box indicates zoomed time-lapse sequential TIRF images which show a dynamic alternation of filopodia growth and retraction as indicated by the white arrows. Scale bars 25 μm. (B) Overexpression of Myo1f-GFP enhances the adhesion of 293T cells on fibronectin-coated plates as assessed by a MTS assay at the indicated time points. This effect was significantly counteracted by co-expression of Tasp1-mCherry. Error bars show the SD (n = 3), asterisks indicate significant differences in mean comparison by two-Way ANOVA(∗: p ≤ 0.05; ∗∗∗∗: p ≤ 0.0001). (C) Overexpression of Myo1f-GFP significantly increases the migration capacity of 293T cells as assessed by a Boyden chamber assay. Error bars show the SD (n = 3), asterisks indicate significant differences evaluated by two-Way ANOVA (∗∗: p ≤ 0.01, ∗∗∗∗: p ≤ 0.001).

Migrating cells often rely on filopodia to sense the environment and to support cell adhesion and movement, thus enabling guided cell migration. Indeed, Myo1f-GFP containing filopodia of transfected HeLa cells are actively involved in probing the environment as sequential TIRF images demonstrate a dynamic alternation of extending and retracting Myo1f-GFP containing filopodia (Figure 5A). Next, we examined whether the Myo1f-induced filopodia alter the cell adhesion capacity. Myo1f-GFP expressing 293T cells adhered more efficiently to fibronectin-coated cell culture wells than non-transfected control cells. Co-expression of proteolytically active Tasp1-mCherry clearly abrogated this effect (Figure 5B). Furthermore, cell motility of Myo1f-GFP expressing cells was determined with a cell migration assay in collagen-coated Boyden chambers with 8 μm pore size. Migratory cells moved towards the chemoattractant in the lower compartment and adhered to the bottom side of the membrane. Here again, significantly more Myo1f-GFP expressing cells migrated through the pores compared to non-transfected cells or cells that co-express Tasp1-mCherry (Figure 5C). These data thus confirm our hypothesis that Myo1f-induced cell protrusions facilitate increased adhesion and expand the migratory capacity of 293T cells. Moreover, concurrent Tasp1 expression antagonizes this effect, supposedly by proteolytic removal of the ATP binding site, which is a part of the short N-terminal cleavage fragment. Even though the C-terminal cleavage fragment still contains the actin-binding site (Figure 1C), the generation of force and motion essentially requires ATP binding.

### A potential role of the myo1f/Tasp1-axis during immune cell development

**Figure 6:**
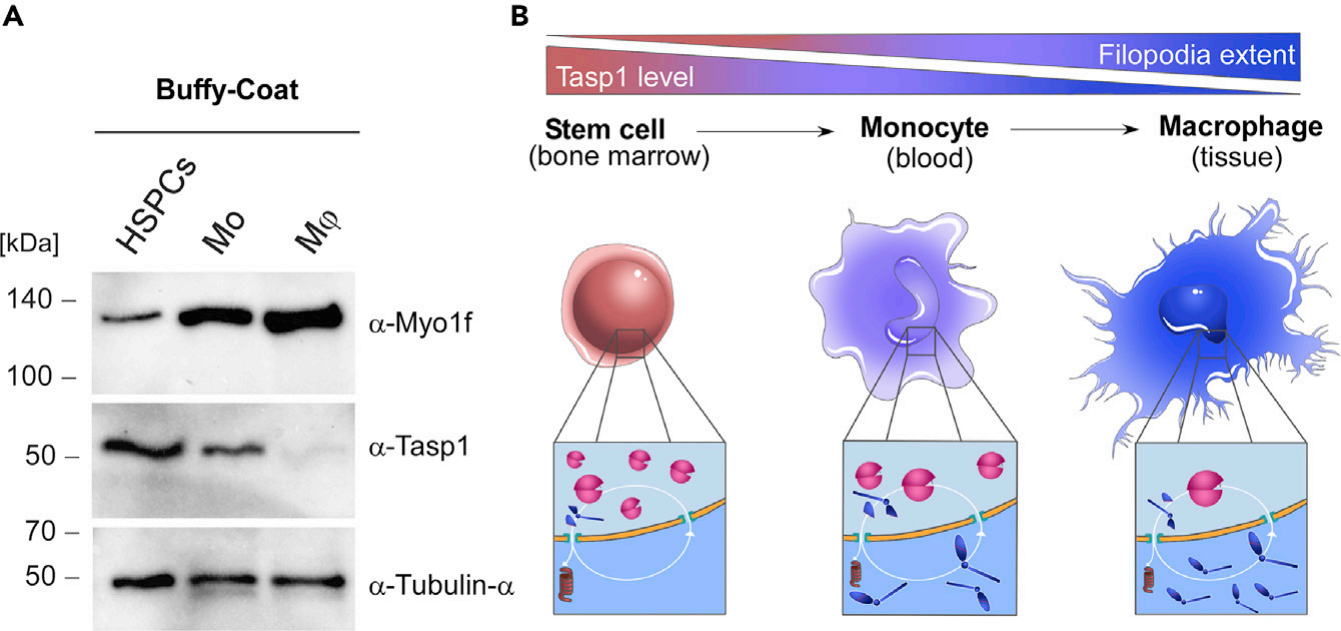
The Myo1f/Tasp1-axis enables regulation of filopodia formation during immune cell differentiation (A) Reduced Tasp1 expression coincides with increased full-length Myo1f concentration during the differentiation of hematopoietic stem and myeloid progenitor cells to monocytes and finally into macrophages. Hematopoietic stem and progenitor cells (HSPCs) and monocytes (Mo) were isolated from human buffy coats. Moreover, isolated monocytes were differentiated into macrophages (Mφ) and the different immune cell fractions were analyzed by immunoblot, α-Tubulin served as a loading control. (B) Model of Myo1f/Tasp1-regulated filopodia formation during macrophage development: filopodia formation increases during hematopoiesis, while Tasp1 levels decline with a higher state of cellular differentiation. Hematopoietic stem and progenitor cells (HSPCs) in the bone marrow contain high Tasp1 levels, resulting in Myo1f-cleavage and degradation. This in turn might suppress Myo1f-induced filopodia formation and associated migration properties (left). Monocytes have emigrated from the bone marrow into the peripheral blood upon appropriate stimuli. A reduction in Tasp1 concentration allows the increase of Myo1f-provoked filopodia (middle). Transendothelial migration from the circulation into tissues promotes monocyte differentiation into macrophages. The characteristic filopodia phenotype of activated macrophages might be facilitated by uncleaved Myo1f (right). See also Figure S5.

As Myo1f is found to be mostly expressed in innate leukocytes, we next compared different types of blood cells isolated from human peripheral blood mononuclear cells (PBMCs). In particular Lin^–^ cells, representing an enriched stem cell fraction, were obtained by depletion of cells that express lineage markers. These enriched stem cells were compared to CD14^+^CD16^–^ cells and macrophages. CD14^+^CD16^–^ cells represent classical monocytes, whereas macrophages were generated from isolated human monocytes by human serum-induced differentiation. Blood cell differentiation occurs by a stepwise developmental progression from hematopoietic stem cells towards lineage commitment. The monocyte-macrophage lineage represents one differentiation pathway in hematopoiesis, where HSPC-derived monocytes are precursors of macrophages and dendritic cells. Interestingly, differentiation from hematopoietic stem and progenitor cells (HSPCs) to monocytes and finally macrophages was indeed accompanied by decreased Tasp1 expression coinciding with increased concentrations of full-length Myo1f in the more specialized cell types (Figures 6A and S5).

This again strongly supports that the inverse correlation of Tasp1 and Myo1f concentration and respectively Myo1f-induced filopodia is also detectable in the physiological context of immune cell differentiation. We conclude that Tasp1 counteracts the filopodia formation capability of Myo1f in hematopoietic stem cells and macrophage precursors and we thus suggest the following model (Figure 6B): During hematopoiesis, full-length Myo1f levels increase while Tasp1 levels decline with a higher state of cellular differentiation. HSPCs reside in the bone marrow, where high Tasp1 expression enables suppression of Myo1f-induced filopodia formation. This allows to retain hematopoietic stem cells in the bone marrow until developmental signals induce their mobilization, and the emanating monocytes start circulating in the bloodstream. The intermediate developmental stage requires active cell protrusions, yet less than terminally differentiated macrophages which rely on filopodia for ECM- and tissue-invasion as well as phagocytosis. In sum, Tasp1-mediated proteolysis might represent a fine-tuning mechanism to modulate the biological function of Myo1f by increasing filopodia length and density during immune cell differentiation.

## Discussion

The unconventional Myo1f was suggested as a potential, previously unknown substrate in a genome-wide bioinformatic screen for the human Tasp1 degradome (Bier et al., 2011b). Our study experimentally verified Myo1f as a substrate of Tasp1. Myo1f cleavage by Tasp1 results in the removal of the anterior 245 amino acids of the N-terminal motor domain containing the ATP-binding site. The resulting main cleavage fragment (Myo1f 246–1098) is strongly destabilized and destined for degradation.

Myo1f is the first confirmed substrate of Tasp1 with a predominant subcellular localization outside the nucleus. The apparent contradiction that a nuclear/nucleolar protease cleaves a cytosolic and plasma membrane-associated protein was resolved by our finding that Myo1f is a nucleo-cytoplasmic shuttle protein equipped with a functional nuclear localization sequence and multiple potential nuclear export signals. Moreover, Myo1f can be added to the growing group of nuclear myosins which have been attributed to functions such as transcriptional activation and relocation of chromatin (Philimonenko et al., 2004; Kulashreshtha et al., 2016).

A contribution of Myo1f to such nuclear key processes is indeed conceivable as we demonstrated an association of Myo1f with RNA-polymerase II and histones. Inside the nucleus, Myo1f might facilitate transcription of genes that are complementary to its pro-migratory function at the cell membrane, although this has to be elucidated in future studies.

In addition, our study unravels that the expression of Myo1f is associated with a massive formation of F-actin rich, cellular membrane protrusions exhibiting characteristic features of filopodia. Successful co-immunoprecipitation of Myo1f and a constitutively active form of Cdc42 further indicates an involvement of Myo1f in filopodia formation. Interestingly, Myo1f-induced filopodia formation was accompanied by increased substrate adhesion and improved migration ability. This is in agreement with previous findings revealing that Myo1f mediates neutrophil migration (Kress et al., 2007; Salvermoser et al., 2018) and with data showing that mouse Myo1f enhances intercellular adhesion capability of macrophages, thereby triggering M1 polarization (Piedra-Quintero et al., 2018).

We thus hypothesize that the unconventional Myo1f executes a specific function to promote the formation of filopodia in the immune response, as Myo1f expression was reported to be particularly elevated in natural killer cells, macrophages, dendritic cells and neutrophils (Kim et al., 2006).

Further, we could demonstrate an inverse correlation between the abundance of filopodia and the amount of cellular Tasp1. The ability of Myo1f to induce, stabilize or elongate filopodia and the concomitant effects of increased cell adhesion and migration are significantly reduced upon Tasp1 co-expression. Indeed, this inverse correlation could be causally attributed to Tasp1-mediated cleavage of Myo1f. Proteolytic processing removes the N-terminal ATP-binding site, rendering the main cleavage fragment incapable of inducing or elongating filopodia. Although the actin-binding site is retained in the C-terminal fragment, ATP binding and hydrolysis are prerequisite to generate force and thus motion (Muretta et al., 2015). Hence, Tasp1-mediated proteolysis is expected to pivotally impact Myo1f functionality and stability.

Different studies have demonstrated that the initiation of filopodia formation in cancer cells from solid tumors leads to an increased migration ability of the transformed cells and accelerated metastatic processes (Gupton and Gertler, 2007; Jacquemet et al., 2015). At first glance, our data seemingly conflict with prior findings, namely that the cleavage of Myo1f by the cancer-promoting Tasp1 prevents Myo1f-induced filopodia formation, which would admittedly avert the acceleration of metastatic processes.

However, Myo1f is predominantly expressed in cells of the innate immune response, therefore the observed effects have to be evaluated in the context of leukocyte development and leukemia. Innate immune cells patrol in the blood and migrate to sites of infection or inflammation in response to certain stimuli. To efficiently migrate in the extracellular matrix and to invade tissues, they rely on filopodia as these structures sense the environment (Davenport et al., 1993) and mediate attachment to the ECM via filopodia tips and shafts which contain adhesion molecules such as integrins and cadherins (Letourneau and Shattuck, 1989; Zhang et al., 2004).

Neutrophils, macrophages and dendritic cells are moreover phagocytic cells which ensure efficient removal of pathogens and other potentially harmful cells. Here, the formation of filopodia is particularly important, since filopodia support the physical capturing of pathogens and mediate the contact to infected or cancerous cells (Kress et al., 2007).

An impaired Myo1f-induced filopodia formation, e.g., resulting from higher Tasp1 expression, might easily disturb the ability of white blood cells to adhere to ECM and to enter tissue and thus even enhance the cancerous potential of white blood cells. As Tasp1 is indeed overexpressed in many leukemic cells (Takeda et al., 2006), its newly discovered Myo1f cleavage ability may deliver a further explanation for the relevance of Tasp1 in cancerogenesis.

Moreover, *TASP1* is a disease-related gene as human *TASP1* gene defects unambiguously result in developmental disorders and congenital immunodeficiency (Suleiman et al., 2018, 2019; Balkin et al., 2019). The clinical phenotype of infants with homozygous deletion or homozygous loss-of function mutations of the *TASP1* gene includes developmental delay, anomalies related to skeletal and organ systems, recurrent respiratory infections, anemia and a lack of early erythroid precursors combined with an impaired megakaryopoiesis. A comparable clinic-pathologic pattern was observed for Tasp1 deficient mice. Besides massive early postnatal lethality, Tasp1^−/−^ animals are smaller in size and display profound skeletal abnormalities coupled with an impaired cell proliferation and a decrease in thymocytes and hematopoietic stem cells (Takeda et al., 2006; Niizuma et al., 2015, 2021). Those observations are consistent with the results of our study, as we provide first evidence that Tasp1 activity might play a role in developmental processes of immune cells. We detected the most prominent Tasp1 levels in an enriched stem cell fraction, whereas the amounts of Tasp1 are decreased in monocytes and further reduced in terminally differentiated macrophages. Inversely, in mature macrophages we detected substantially higher amounts of full-length Myo1f protein, correlating with an augmented filopodia formation. It is thus tempting to speculate that Tasp1-mediated cleavage of Myo1f might enable the adjustment of filopodia quantity during the development of macrophages, and maybe also of neutrophils and dendritic cells. However, this correlation must be validated in comprehensive functional studies in different immune cell populations to experimentally prove a causative role of Tasp1.

In conclusion, Tasp1-mediated proteolytic cleavage might serve as a fine-tuning mechanism to regulate cellular Myo1f concentration and activity post-transcriptionally, thereby dynamically modulating filopodia density and length, e.g., essential during differentiation of innate immune cells.”

### Limitations of the study

Some of our observations were primarily based on Myosin1f overexpression studies.

To overcome this limitation we tried to perform Myosin1f and Taspase1 loss-of-function studies in immune cells but we observed transfection efficiencies of monocytes and macrophages less than 10% reflected by a minimal reduction of respective protein expression levels in Myo1f-siRNA and Tasp1-siRNA transfected cells compared to the non-silencing control siRNA approach. A meaningful comparison of filopodia content was therefore not possible. However, we were able to demonstrate in an alternative experimental approach that endogenous Myosin1f indeed fulfills a physiological function in filopodia formation: untagged, endogenous Myosin1f was visualized by immunofluorescence microscopy with a Myosin1f antibody in THP-1 monocytes and PMA-differentiated THP-1 macrophages. Endogenous Myosin1f was primarily detected in the filopodia of macrophages, and thus resembled the localization pattern observed for overexpressed Myosin1f-GFP.

Although we were able to demonstrate that Myosin1f enhances cellular filopodia level, the underlying mechanism remains to be determined. Moreover, future studies are needed to elucidate the exact function of nuclear Myo1f.

## STAR★Methods

### Key resources table

**Table:** 

REAGENT or RESOURCE	SOURCE	IDENTIFIER
**Antibodies**		

Mouse monoclonal anti-GFP (B-2)	Santa Cruz Biotechnology	Cat#: sc-9996; RRID: AB_627695
Mouse monoclonal anti-His	Quiagen	Cat#: 34660; RRID: AB_2619735
Rabbit polyclonal anti-HA tag	Abcam	Cat#: ab9110; RRID: AB_307019
Rabbit polyclonal anti-Taspase1	Abcam	Cat#: ab63160; RRID: AB_1143330
Mouse monoclonal anti Myosin1f	Santa Cruz Biotechnology	Cat#: sc-376534; RRID: AB_11150122
Mouse monoclonal anti-Tubulin α	Sigma	Cat#: T6074; RRID: AB_477582
Mouse monoclonal anti-RNA Polymerase II/POLR2A	Novus Biologicals	Cat#: NB200-598; RRID: AB_2167465
Mouse monoclonal anti-Histone H3	Abcam	Cat#: ab195277; RRID: AB_2885180
Sheep anti-mouse IgG, HRP-linked	GE Healthcare	Cat#: NXA931; RRID: AB_772209
Donkey anti-rabbit IgG, HRP-linked	GE Healthcare	Cat#: NA934; RRID: AB_772206
Donkey anti-goat IgG, HRP-linked	Santa Cruz Biotechnology	Cat#: sc-2033; RRID: AB_631729
Goat anti-mouse IgG (H + L), Superclonal Recombinant Secondary Antibody, Alexa Fluor 488	Thermo Fisher Scientific	Cat#: A28175; RRID: AB_2536161

**Bacterial and virus strains**		

BL21-CodonPlus (De3)-RIL Competent Cells	Agilent Technologies	Cat#: 230245;

**Biological samples**		

buffy coats from anonymous healthy donors	Institute of Transfusion Medicine, University Hospital Essen	N/A

**Chemicals, peptides, and recombinant proteins**		

Invitrogen™ Lipofectamine™ 2000 Reagent	Fisher Scientific	Cat#: 10696153
PMA (phorbol 12-myristate 13-acetate)	Sigma-Aldrich	Cat#: P1585; CAS: 16561-29-8
Leptomycin B (LMB)	Sigma-Aldrich	Cat#: L2913; CAS: 87081-35-4
Bortezomib	Calbiochem	CAS: 179324-69-7
Roti®-Histofix 4 %	Roth	Cat#: P087.1
Phalloidin-Tetramethylrhodamine B	Sigma-Aldrich	Cat#: P1951
Protease inhibitors (Complete Mini EDTA-free cocktail tablets)	Roche	Cat#: 04693124001
Pierce™ ECLplus Western Blotting Substrate	Thermo Fisher Scientific	Cat#: 32132
Hoechst 33342	Thermo Fisher Scientific	Cat#: H3570; CAS: 23491-52-3

**Critical commercial assays**		

Millipore QCM™ Haptotaxis Cell Migration kit	Sigma-Aldrich	Cat#:ECM580
Cell titer ® AQueous One	Promega	Cat#: G3582
Chromatin Extraction Kit	Abcam	Cat#: ab117152
μMACS GFP Isolation Kit	Miltenyi Biotec	Cat#: 130-091-125
Classical Monocyte Isolation Kit human	Miltenyi Biotec	Cat#:130-117-337
Diamond CD34 Isolation Kit	Miltenyi Biotec	Cat#:130-094-531

**Experimental models: Cell lines**		

H.sapiens: HEK 293T	ATCC	CRL-3216; RRID: CVCL_0063
H.sapiens: HeLa Kyoto	from S. Narumiya, Kyoto University	RRID: CVCL_1922
H.sapiens: SW480	ATCC	CCL-228; RRID: CVCL_0546
H.sapiens: THP-1	ATCC	TIB-202; RRID: CVCL_0006

**Oligonucleotides**		

Forward primer for coding region amplification of the N-terminal Tasp1 cleavage fragment, comprising amino acids 1 to 245 of Myo1f: TTTGGTACCGGCAGCAAGGAGGCTTCCAC	This paper	N/A
Reverse primer for coding region amplification of the N-terminal Tasp1 cleavage fragment, comprising amino acids 1 to 245 of Myo1f: TTTGCTAGCGTCCACCTGGTAGGTGTC	This paper	N/A
Forward primer for coding region amplification of the C-terminal Tasp1 cleavage fragment, comprising amino acids 246 to 1098 of Myo1f: AAAGGTACCGGCACGGACGACAGAAG	This paper	N/A
Reverse primer for coding region amplification of the C-terminal Tasp1 cleavage fragment, comprising amino acids 246 to 1098 of Myo1f: TTTGCTAGCGATCTTCTCCACGTAGTTTCCTGG	This paper	N/A
Forward annealed oligonucleotide for N-terminal myc epitope tag insertion: GATGGAACAAAAACTTATTTCTGAAGAAGATCTGGGTAC	This paper	N/A
Reverse annealed oligonucleotide for N-terminal myc epitope tag insertion: CCAGATCTTCTTCAGAAATAAGTTTTTGTTCCATCGTAC	This paper	N/A

**Recombinant DNA**		

Plasmid: pcDNA3.1-GFP	Laboratory of Roland Stauber; Bier et al. (2011b)	N/A
Plasmid: pc3-Tasp1-mCherry	Knauer et al. (2011)	N/A
Plasmid: pc3-Tasp1-HA	Bier et al. (2011a)	N/A
Plasmid: pc3-TaspT234V-HA	Bier et al. (2011a)	N/A
Plasmid: pc3-Myo1f-GFP	Bier et al. (2011b)	N/A
Plasmid: pc3-Myo1f -^245^AA^246^-GFP	This paper	N/A
Plasmid: pET-22b Tasp1-His	Knauer et al. (2011)	N/A
Plasmid: pcDNA3-mCherry-Cdc42 wt	Laboratory of Perihan Nalbant; Nalbant et al. (2004)	N/A
Plasmid: pcDNA3-mCherry-Cdc42 Q61L	Laboratory of Perihan Nalbant; Nalbant et al. (2004)	N/A
Plasmid: pc3-myc-Myo1f-N-term-GFP	This paper	N/A
Plasmid: pc3-myc-Myo1f-C-term-GFP	This paper	N/A

**Software and algorithms**		

GraphPad Prism 9	https://www.graphpad.com/	RRID: SCR_002798
Fiji (ImageJ)	Schindelin et al. (2012)	RRID: SCR_003070; https://ImageJ.nih.gov/ij/
CellProfiler3	McQuin et al. (2018)	https://cellprofiler.org/
AxioVision software	https://www.micro-shop.zeiss.com/de/de/system/software	version 4.7
cNLS mapper	Kosugi et al. (2009)	http://nls-mapper.iab.keio.ac.jp/cgi-bin/NLS_Mapper_form.cgi
NES Finder 0.2	http://research.nki.nL/fornerodlab/NES-Finder.htm	N/A
LocNES	Xu et al. (2015)	http://prodata.swmed.edu/LocNES/LocNES.php
FiloQuant, Filopodia quantification plugin	Jacquemet et al. (2017)	https://github.com/guijacquemet/FiloQuant

### Resource availability

#### Lead contact

Further information and requests for resources and reagents should be directed to and will be fulfilled by the lead contact, Astrid Hensel (astrid.hensel@uni-due.de).

#### Materials availability

All plasmids generated in this study are available from the lead contact without restriction.

#### Data and code availability

This study did not generate any unique datasets. All data generated during this study are either supplied in the figures and the supplemental information or will be shared by the lead contact upon request. This paper does not report original code.
Any additional information required to reanalyze the data reported in this paper is available from the lead contact upon request.

### Experimental model and subject details

#### Cell lines

HEK 293T cells (donor: female fetus), SW480 cells (donor: 50-year-old human male) and THP1-cells (donor: 1-year-old human male) were obtained from the American Type Culture Collection (ATCC). HeLa Kyoto cells (donor: 30,5-year-old human female) were obtained from S. Narumiya, Kyoto University. Cell lines were cultivated at 37°C in a relative humidity of about 95% and 5% CO_2_. HEK 293T- and HeLa-cells were maintained in Dulbecco’s Modified Eagle’s Medium (DMEM) supplemented with 10% (v/v) fetal calf serum (FCS), 2 mM L-Glutamine and 1% (v/v) Gibco® antibiotic-antimycotic and split at a confluency of about 70%–90%. SW480 and THP-1 cells were grown in RPMI-1640 medium supplemented with the components stated above and for THP-1 cultivation additionally 0.05 mM 2-mercaptoethanol were added. THP-1 monocyte differentiation was induced with 100 nM PMA (phorbol 12-myristate 13-acetate) treatment for three days.

#### Human buffy coats

Buffy coats from anonymous healthy donors, who gave their written informed consent, were provided from the Institute of Transfusion Medicine of the University Hospital Essen. According to German law, the use of anonymized buffy coats for scientific research does not require specific ethical approval.

### Method details

#### Cloning

Eukaryotic expression constructs encoding wild-type Tasp1 and a cleavage-deficient mutant C-terminally tagged with mCherry and HA were described previously (Bier et al., 2011a) as well as the vector pET-22b encoding Tasp1-His for bacterial expression (Knauer et al., 2011). Plasmids pc3-Myo1f-GFP and pc3-Myo1f -^245^AA^246^-GFP are based on a Myo1f coding sequence amplified from human head and neck tumor cDNA, and cloned into the expression vector pc3-GFP as described (Bier et al., 2011b).

The coding region of the N-terminal Tasp1 cleavage fragment, comprising amino acids 1 to 245, was amplified from plasmid pc3-Myo1f-GFP, as well as the C-terminal part of Myo1f-GFP downstream of the Tasp1 cleavage site, comprising amino acids 246 to 1098 (primer sequences are depicted in the key resources table). PCR products were cloned into the vector pcDNA3.1-GFP via KpnI/NheI. Both resulting constructs were additionally equipped with a N-terminal myc epitope tag by insertion of annealed oligonucleotides via KpnI as described (Bier et al., 2011b), resulting in the eukaryotic expression plasmids pc3-myc-Myo1f-N-term-GFP and pc3-myc-Myo1f-C-term-GFP. All plasmids were finally confirmed by restriction enzyme digestion and sequencing. Plasmids pcDNA3-mCherry-Cdc42 wt and pcDNA3-mCherry-Cdc42 Q61L were derived from the corresponding GFP-tagged constructs described previously (Nalbant et al., 2004).

#### Transfection and treatment of mammalian cells

Cells were transfected using Lipofectamine® 2000 (Thermo Fisher Scientific) according to the manufacturer’s protocol. For proteasome inhibition, 293T cells were transiently transfected with the indicated plasmids and 18 h later treated with 50 nM bortezomib for 6 h. Treatment with the general export inhibitor Leptomycin B (LMB) was conducted 24 h after transient transfection in a concentration of 5 nM for 3 h.

#### Cell fixation, microscopy and imaging

HEK293T-, HeLa- and SW480 cells were seeded in ibiTreat μ-slide 8-well chamber slides (ibidi®) and transfected with Lipofectamine® 2000 or left untreated. After 24 h, cells were washed with PBS and fixed with Roti®-Histofix 4% (4% phosphate buffered formaldehyde solution; Roth) for 20 min at room temperature. Cells were washed with PBS and stained with Hoechst33342 (10 mg/mL in H_2_O, 1:1000) for 15 min in DPBS (1% BSA, 0.3% Triton) or with Phalloidin-Tetramethylrhodamine B (Sigma-Aldrich) for 45 min. Before incubation with the staining reagent, fixed cells were incubated with 0.1% Triton X-100 in PBS for 15 min to increase permeability.

Confocal laser scanning microscopy of fixed cells was performed on a TCS SP8X Falcon Confocal Microscope (Leica Microsystems) using the HC PL APO 63×/1.2 W motCORR CS2 water-immersion objective. The laser lines of the white light laser used for excitation were 488 nm (EGFP) and 561 nm (mCherry and rhodamine). Fluorescence was detected using HyD detectors (490–540 nm) and (575–650 nm), respectively. Images were acquired using the “LAS X” software (Leica Microsystems) and assembled with “Canvas 5” (ACD Systems).

For live cell imaging HeLa cells were seeded on 35mm glass bottom dishes coated with fibronectin (10 μg/mL, Corning) and transfected as mentioned above. Live cell TIRF and spinning disc confocal images were then acquired with an Eclipse Ti-E8Nikon) inverted microscope with TIRF illuminator Unit, an AOTC Laser Combiner, a laser dual spinning disc scan Head (CSU-X1: Yokogawa), and an iXon3 897 single-photon-detection EMCCD camera. Laser lines 488 and 561 nm were used. Images were acquired using an Apo TIRF 60×/1.49 NA oil immersion objective with EM gain of 50–100 and 1∗1 binning. A CSU Quad Dichroic mirror set and TIRF Dual line Beamsplitter zt 488/561 rpc were used. Acquisition was controlled with Andor IQ Software and images were processed using ImageJ.

#### Immunoprecipitation

293T cells were harvested 24 h after transfection in a low salt lysis buffer (50 mM Tris pH 8.0, 150 mM NaCl, 5 mM EDTA, 0.5% NP-40, 1 mM DTT, 1 mM PMSF and a cOmplete protease inhibitor cocktail tablet from Roche Diagnostics) at 4°C. After two sonification steps (20 s at 95% amplitude) with a Sonopuls mini20 device (Bandelin), cell debris were removed (15,000 g, 25 min, 4°C) to obtain whole cell lysates for immunoprecipitation. Alternatively, chromatin extract fractions were prepared from 293T cells with the Chromatin Extraction Kit (Abcam) according to the manufacturer’s instructions.

Co-immunoprecipitations were performed using μMACS anti-GFP Micro-Beads and μ-MACS (magnetic antibody cell sorting) columns (Miltenyi Biotec) according to the suppliers’ recommendation. Briefly, whole cell lysates or chromatin fractions were incubated with 50 μL μ-MACS anti-GFP Micro-Beads for 30 min on ice. μm-MACS columns were placed into the magnetic field stand and equilibrated with 200 μL lysis buffer. Lysates with magnetic beads were applied onto the columns, washed three times with 200 μL lysis buffer, one time with 200 μL wash buffer-1 and once with 100 μL wash buffer-2 (Miltenyi Biotec). Afterwards, 20 μL pre-heated elution buffer (Miltenyi Biotec) were applied onto each column and incubated for 5 min 50 μL pre-heated elution buffer were applied to each column for complete elution. The eluates, as well as input samples of the lysates (or chromatin extract) were subjected to SDS-PAGE and immunoblotting.

#### Isolation of human immune cells

For isolation of PBMCs, buffy coats from anonymous healthy donors, who gave their written informed consent, were provided from the Institute of Transfusion Medicine of the University Hospital Essen. According to German law, the use of anonymized buffy coats for scientific research does not require specific ethical approval. The buffy coats were diluted 1 : 1 in non-complemented RPMI-medium and were slowly layered above Ficoll-Paque (Cytiva), which was placed in 50 mL conical tubes. After density gradient centrifugation at room temperature, 760 × g, for 20 min (with the breaks OFF), the layer of PBMCs at the interface between plasma- and Ficoll-layer was collected and washed three times with 40 mL PBS via centrifugation at room temperature, 350 × g, for 8 min. A fourth centrifugation step at 200 × g, for 10 min was conducted to achieve platelet removal. Subsequently, PBMCs concentration was determined by cell counting. Monocytes were isolated in by negative selection with the “Classical Monocyte Isolation Kit human” (Miltenyi Biotec) according to the manufacturer’s instructions. Lin^−^ cells that represent enriched stem cells were isolated from human buffy coat-derived PBMCs with the “Diamond CD34 Isolation Kit” (Miltenyi Biotec), by performing only the first step of the two-step procedure: Via magnetic separation Lin^+^ cells were removed from the PBMCs after being labeled with a mixture of biotin-conjugated antibodies against lineage specific marker antigens. Hematopoietic stem cells, which do not express mature blood cell markers were enriched in the flow-through fraction (Lin^−^ cells). The second part of the kit protocol, the subsequent positive selection of CD34^+^ stem cells, was not performed as the expected yield of pure CD34^+^ stem cells are extremely low.

#### RIPA cell lysis

Cells were harvested in RIPA lysis buffer (20 mM Tris-HCl pH 8, 137 mM NaCl, 2 mM EDTA and 1% NP-40) with protease inhibitor cocktail at 4°C. After two sonification steps, cell debris was removed and protein concentrations were determined and adjusted using the Bio-Rad protein assay system (Bio-Rad Laboratories). Equal loading of lysates was controlled by using Tubulin α-antibody as a loading control.

#### Immunoblotting

Immunoprecipitates, total protein lysates, chromatin extracts and cleavage assay samples were separated by SDS–PAGE and subsequently transferred to polyvinylidene difluoride (PVDF) membranes (Amersham Hybond, GE Healthcare) via the PerfectBlue™ tank electro blotter (Peqlab). After incubation with blocking buffer (5% milk in Tris-Buffered Saline, 0.1% Tween (TBS-T)), membranes were probed with the respective primary antibodies (see key resources table) and incubated overnight at 4°C. After washing five times with TBS-T, HRP-conjugated secondary antibodies (listed in key resources table) were applied for 1h at RT, subsequently followed by five washing steps. Immunoconjugations were detected with the ECLplus Western Blotting Substrate (Pierce) and the ChemiDoc MP Imaging System (Bio-Rad Laboratories GmbH).

#### Immunofluorescence

Differentiation of THP-1 cells into macrophages was induced with 100 nM PMA (phorbol 12-myristate 13-acetate) treatment for four days. After 2 × 5 min washing with PBS, cells were fixed with 4% paraformaldehyde in PBS for 20 min at RT. Following two further washing steps, cells were blocked and permeabilized in 0.3% Triton X-100, 5% goat serum in PBS for 30 min at RT and incubated afterwards with anti-Myo1f antibody (diluted 1:100 in 0.3% Triton X-100, 1% BSA in PBS) in a humidified chamber overnight at 4 °C. After 2 × 5 min washing with PBS, cells were incubated for 2 h with the fluorophore coupled secondary antibody (goat-*anti*-mouse-Alexa488 goat, Invitrogen), diluted 1:1000 in PBS containing 0.3% Triton X-100, 1% BSA in a humidified chamber protected from light at RT, and subsequently washed 2 times with PBS. The cells were incubated with Phalloidin-Tetramethylrhodamine B (35545A, Sigma-Aldrich), diluted 1:1000 in DPBS for 45 min at RT and finally washed 2 times with PBS.

Non-adherent THP-1 monocytes (1 × 10^6^ cells) were centrifuged (500 × g, 5 min, 4°C), media was aspirated and the immunostaining steps and solutions were exactly the same as described above. After each incubation step, a centrifugation at 500 × g was necessary to separate and remove supernatant. After last washing steps, centrifuged cells were resuspended in 100 μL PBS, 50 μL of this suspension were dropped on a glass slide. After air-drying the cells were covered with a drop Roti®-Mount Aqua (Roth) and a coverslip.

#### Tasp1-His *semi-in vitro* cleavage assay

For semi*-in vitro* Tasp1 cleavage experiments, His-tagged wild-type Tasp1 was expressed in BL21-CodonPlus (De3)-RIL cells and purified as described before (Knauer et al., 2011). 293T lysates of cells which expressed either Myo1f-GFP or Myo1f-^245^AA^246^-GFP for 24 h were generated and incubated at 37°C with 10 μM Tasp1-His in reaction buffer (100 mM HEPES at pH 7.9, 5 mM MgCl_2_, 20 mM KCl, 10% (w/v) sucrose, 5 mM DTT), or the same volume of buffer only. Equal amounts of the reaction were stopped with 5 × SDS buffer at the indicated time points and were subjected to SDS-PAGE.

#### Cell adhesion and migration assays

For cell adhesion assay, cells were plated in 6-well plates and were transiently transfected with Myo1f-GFP and cleavage mutant Myo1f-GFP 24 h prior to the assay at 50% confluence. Cells were washed, trypsinized (TrypLE™, 0.5 mL), counted and diluted to yield a concentration of 5 × 10^5^ cells/mL. Then cells (100 μL with 5 × 10^4^ cells/well) were plated in collagen I coated 96-well plates (Thermo Fisher Scientific) and incubated (37°C, 5% CO_2_) for the indicated time points. After incubation, medium was aspirated, and non-adherent cells were washed off with PBS. Fresh medium was added, and the viable cells were quantified using the MTS assay. Transwell cell migration assays were performed using the Millipore QCM Haptotaxis Cell Migration kit (Sigma Aldrich), according to the manufacturer’s instructions.

#### MTS-assays

Quantification of adherent cells was performed by measuring viable cells with the MTS (3-(4.5-dimethylthiazol-2-yl)-5-(3-carboxymethoxyphenyl)-2-(4-sulfophenyl)-2H-tetrazolium) assay (Cell titer 96® AQueous One; Promega). After medium was aspirated from the 96-well plates and exchanged against 100 μL fresh medium, 20 μL MTS solution were added per well. Cells were incubated for 3 h at 37°C in 5% CO_2_. The color shift induced by the Formazan metabolite of MTS proportional to the amount of viable cells was recorded on a plate reader (Glomax multi; Promega) at 490 nm absorbance.

#### Bioinformatics

The protein sequence of the unconventional Myo1f (UniProtKB - O00160) was analyzed for the presence of nuclear localization (NLS) and nuclear export signals (NES) with the web-based motif predictor tools “cNLS mapper” (http://nls-mapper.iab.keio.ac.jp/cgi-bin/NLS_Mapper_form.cgi) and “NES Finder 0.2” (http://research.nki.nl/fornerodlab/NES-Finder.htm) and “LocNES” (http://prodata.swmed.edu/LocNES/LocNES.php).

### Quantification and statistical analysis

#### Fluorescence-based quantification of nuclear Myo1f-GFP

Nuclei were counterstained with Hoechst 33342 to define regions of interest (ROI) for analysis with the software “CellProfiler3” (McQuin et al., 2018). Within the ROIs, the mean fluorescence intensity of Myo1f-GFP (500–550 nm) was measured and compared between LMB treated and untreated cells. Statistical evaluation of the differences between the two datasets of every experiment was conducted via t-tests. Additionally, the populations of mean fluorescence intensity of both treated and untreated nuclei were fitted to a Gaussian frequency distribution in order to better visualize the difference between these populations. For every experiment n ≈ 100 treated as well as untreated cells were captured and a total of four independent experiments were performed.

#### Quantification of filopodia

Filopodia of Myo1f-GFP and GFP expressing HeLa cells (Figure 3B) were automatically analyzed and quantified with FiloQuant software (Jacquemet et al., 2017).

Tasp1 influence on Myo1f induced filopodia formation (Figure 4) was analyzed by confocal imaging of a sufficient number of HeLa cells as well as 293T cells (n > 80) expressing Myo1f-GFP or Myo1f-^245^AA^246^-GFP together with or without Tasp1-mCherry. 3D z-stacks of whole cells were acquired and filopodia amount and length was analyzed by manual evaluation using Fiji tools. To ensure unbiased quantification, image file names were randomized using the “Filename_randomizer.txt" macro for ImageJ.

Two classes of filopodia were defined by categorizing filopodia according to length: < 5 μm and > 5 μm. The amount of filopodia smaller than 5 μm seemed to be stable within each Myo1f-GFP expressing cell line and independent of Tasp1 co-expression. Therefore, filopodia longer than 5 μm were quantified and assessed by pairwise comparison via t-test among the cells expressing the same Myo1f variant with or without Tasp1 co-expression.

### Statistical analysis

Statistical analysis was performed with GraphPadPrism 9 software. Statistical relevance of the data was determined by applying unpaired Student’s t-test (Figures 2C, 3B and 4A/B) or Two-Way ANOVA(Figures 5B/C). Further statistical analysis details for each experiment (significance levels, number of samples and repeats) are indicated in the respective figure legends and in the STAR methods. p values <0.05 were considered statistically significant.
