# Supplementary material for: The Taspase1/Myosin1f-axis regulates filopodia dynamics
Source: iScience. 2022 May 5;25(6):104355. doi: 10.1016/j.isci.2022.104355 (PMC9121324; doi:10.1016/j.isci.2022.104355)
Supplement: Document S1. Figures S1–S5 [file mmc1.pdf]

iScience, Volume *25*

Supplemental information

The Taspase1/Myosin1f-axis regulates filopodia dynamics

Astrid Hensel, Paul Stahl, Lisa Moews, Lena König, Rutuja Patwardhan, Alexander Höing, Nina Schulze, Perihan Nalbant, Roland H. Stauber, and Shirley K. Knauer

**Potential NLS in unconventional myosin-If (predicted by “cNLS mapper”)**		
aa sequence	*predicted signal*	**score**
^{36}NLRKRFMDDYIFTYIGSVLISVNPFKQMPY^{65}	bipartite NLS	6
**Potential NES in unconventional myosin-If**		
**predicted by “NES Finder 0.2”**	**predicted by “LocNES“**	
aa sequence	*aa sequence*	**score**
^{256}TLSAMQVIGI^{265}		
^{274}LQLVAGILHL^{283}	^{269}IQQLVLQLVAGILHL^{283}	0.375
^{279}GILHLGNISF^{288}	^{274}LQLVAGILHLGNISF^{288}	0.234
^{296}RVESVDLLAF^{305}		
^{383}GVLDIYGFEI^{392}		
^{410}LQQIFIELTL^{419}	^{405}FVNEKLQQIFIELTL^{419}	0.392
^{601}VKHQVEYLGL^{610}	^{596}WEENRVKHQVEYLGL^{610}	0.179
^{894}FSRGFGDLAV^{903}		
^{897}GFGDLAVLKV^{906}		
^{1053}VGQDVDELSF^{1062}	^{1048}ALYQYVGQDVDELSF^{1062}	0.188

Figure S1. Prediction of potential intracellular transport signals in Myosin1f, related to Figure 2. The protein sequence of the unconventional Myosin1f (UniProtKB - O00160) was analyzed for the presence of intracellular transport signals, in particular nuclear localization (NLS) and nuclear export signals (NES) with the web-based motif predictor tools “cNLS mapper” (http://nls-mapper.iab.keio.ac.jp/cgi-bin/NLS_Mapper_form.cgi), “NES Finder 0.2” (http://research.nki.nl/fornerodlab/NES-Finder.htm) and “LocNES” (http://prodata.swmed.edu/LocNES/LocNES.php), respectively. Whereas only one potential bipartite NLS in the extreme N-terminus of Myosin1f was predicted with “cNLS mapper”, 5 potential nuclear export signals were identified in the C-terminal portion of the protein with the online tool “NES Finder 0.2” and with the algorithm “LocNES“. The calculated scores are indicated in the last column. Briefly, “cNLS mapper” extracts putative NLS sequences with a score equal to or more than the selected cut-off score, ranging between 1 and 10, with a higher score indicating stronger NLS activities. “LocNES“ attributes probability scores ranging between 0 and 1. Amino acid residues included in the “LocNES”, but not the “NES finder 0.2” prediction are marked in grey.

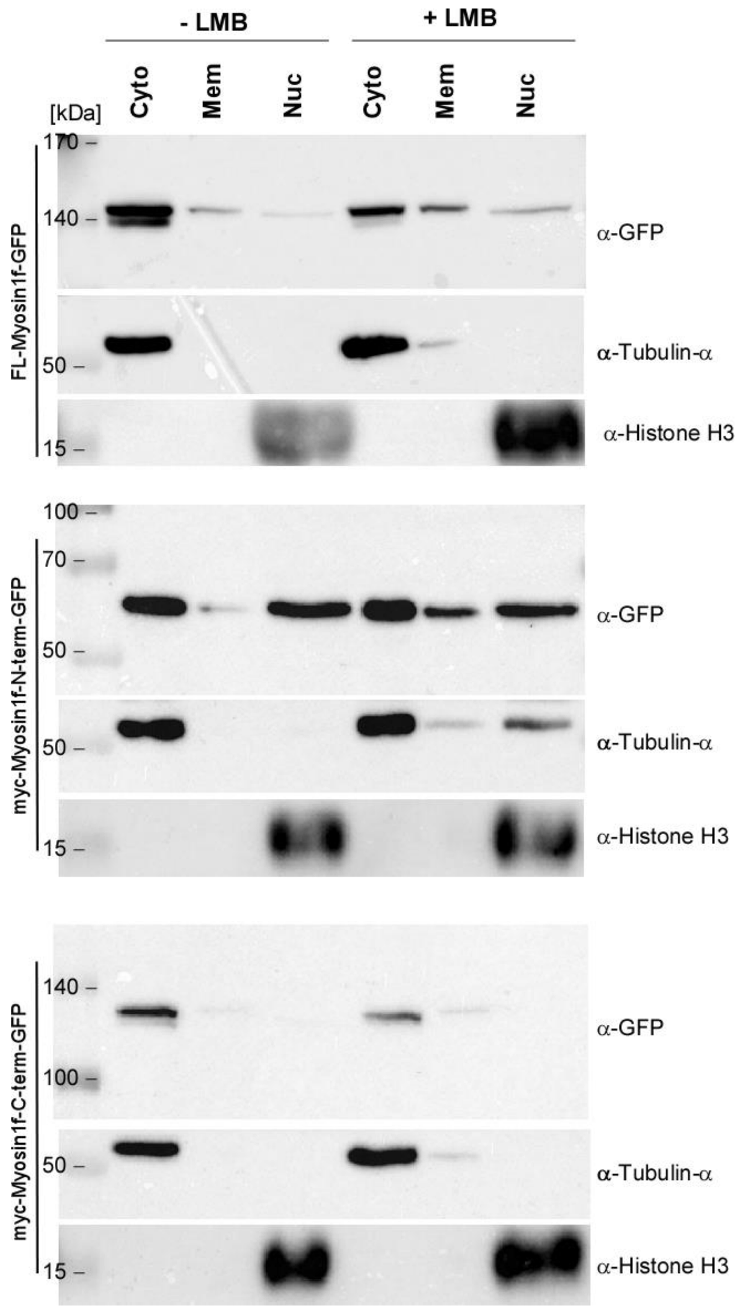

Figure S2. Subcellular distribution of full-length Myosin1f and Myosin1f-truncations representing the N- and the C-terminal Taspase1 cleavage products, related to Figure 2.

293T cells, expressing the indicated Myosin1f-variants were either exposed to 5 nM Leptomycin B (+ LMB) or a mock treatment (- LMB) for 3h and subsequently fractionated using Subcellular Protein Fractionation Kit (Thermo Fisher Scientific). Protein concentration of each fraction was determined and equal protein amounts of cytoplasmic (Cyto), membrane (Mem) and nuclear (Nuc) fractions were analyzed with SDS-PAGE followed by immunoblotting to detect GFP fusion proteins. Nuclear marker Histone H3 and cytosolic Tubulin α confirmed the identity of the subcellular fractions. Full length Myosin1f-GFP is detected almost exclusively in the cytosolic and membrane fraction, but was found to be enriched in nuclear fraction upon LMB treatment. In contrast, a large portion of the N-terminal fragment is detected in the nuclear fraction regardless of LMB treatment, whereas the C-terminal fragment is mainly cytoplasmic.

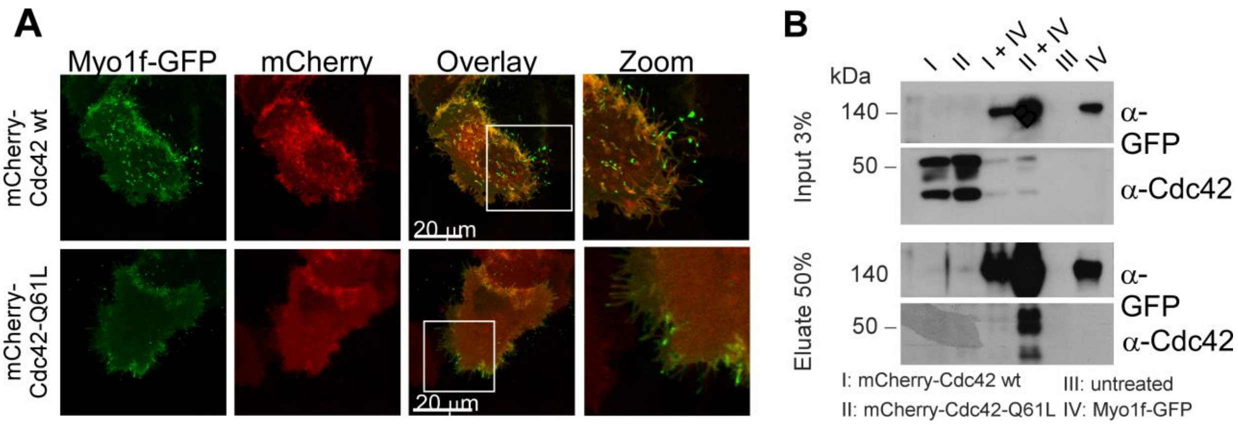

Figure S3. Functional interplay of Myosin1f and Cdc42, related to Figure 3. (A) Colocalization of Myosin1f and Cdc42 variants upon co-expression in HeLa cells for 24 h. Scale bars, 20 µm. (B) Myosin1f preferably interacts with the constitutively active Cdc42-Q61L as compared to the wild-type form. Co-immunoprecipitation of GFP-tagged Myosin1f with indicated Cdc42 variants from 293T cells and detection by immunoblot analysis.

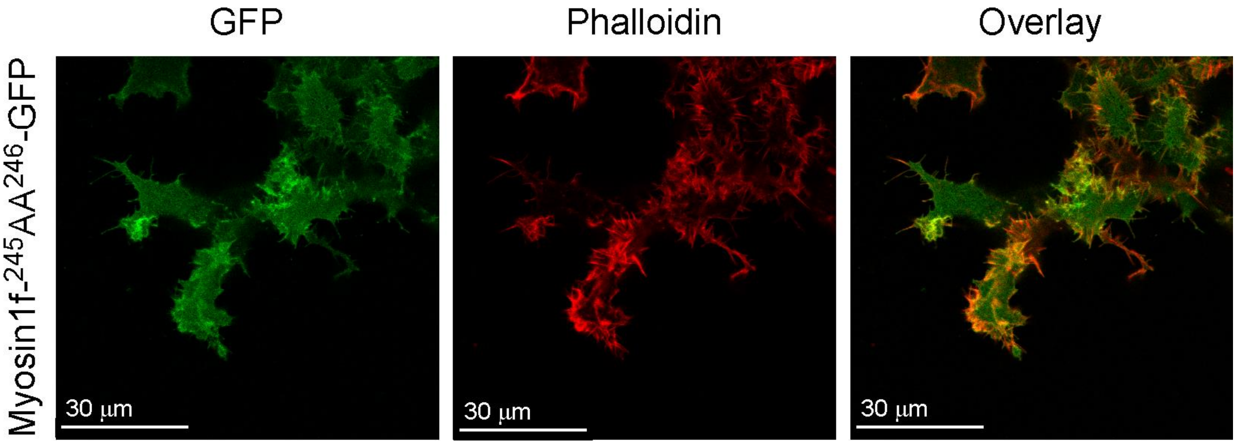

Figure S4. Uncleavable Myosin1f also promotes filopodia formation, related to Figure 3. 293T cells were transfected with a Myosin1f variant with a mutated Taspase1 consensus recognition sequence (Myosin1f-^{245}AA^{246}-GFP) and fixed 24 h later. Filamentous F-actin was stained with Rhodamine-conjugated phalloidin (red) and cells were analyzed by confocal microscopy. Scale bars, 30 µm.

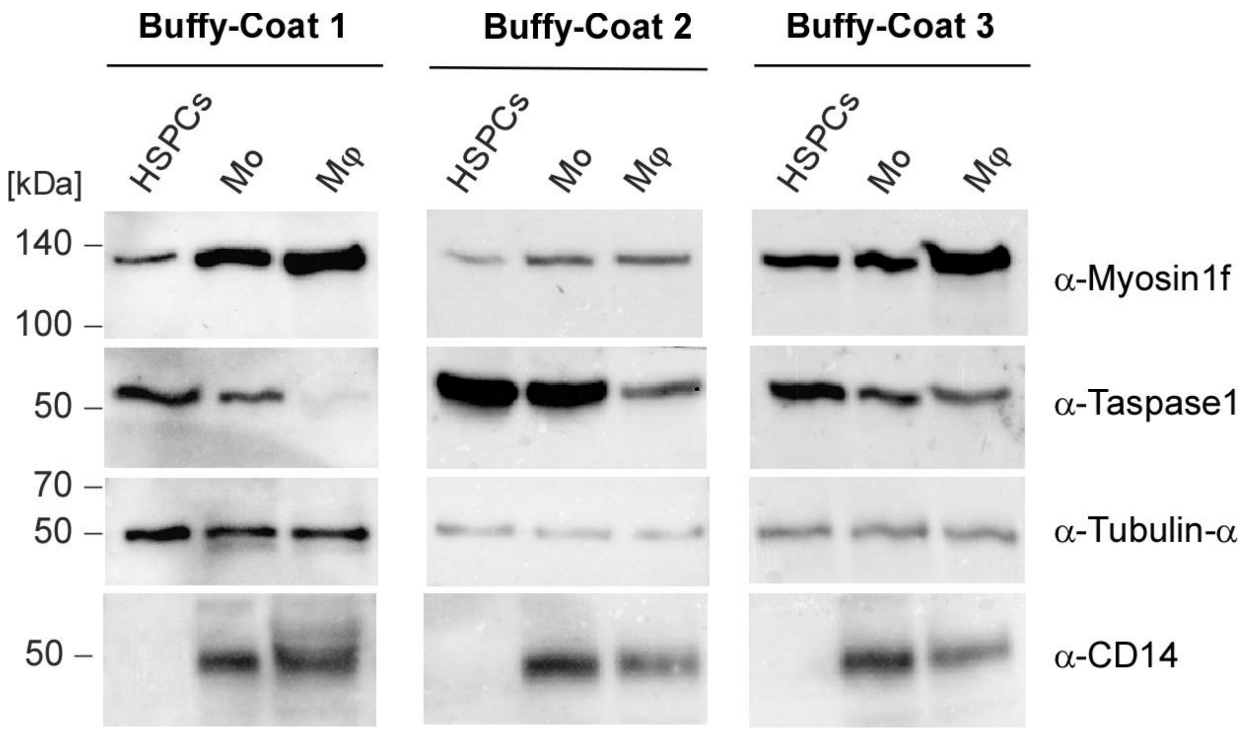

Figure S5. Three independent replicates of immune cell isolation from three buffy coats, related to Figure 6. Reduced Tasp1 expression coincides with increased full-length Myo1f concentration during the differentiation of hematopoietic stem and myeloid progenitor cells to monocytes and finally into macrophages. Hematopoietic stem and progenitor cells (HSPCs) and monocytes (Mo) were isolated from buffy coats. Moreover, isolated human monocytes were differentiated into macrophages (Mφ) and the different immune cell fractions were analyzed by immunoblot, α-Tubulin served as a loading control. Cluster of differentiation 14 (CD14), which is described as monocyte/ macrophage differentiation antigen, was used as a marker for myeloid cells to check identity of isolated immune cell fractions from buffy coats 1-3.
